# Betuletol, a Propolis Component, Suppresses IL-33 Gene Expression and Effective against Eosinophilia

**DOI:** 10.3390/molecules27175459

**Published:** 2022-08-25

**Authors:** Aurpita Shaha, Rezwanul Islam, Naonobu Tanaka, Yoshiki Kashiwada, Hiroyuki Fukui, Noriaki Takeda, Yoshiaki Kitamura, Hiroyuki Mizuguchi

**Affiliations:** 1 Department of Molecular Pharmacology, Institute of Biomedical Sciences, Tokushima University Graduate School, Tokushima 770-8503, Japan; 2 Laboratory of Tumor Microenvironment and Metastasis, The Hormel Institute, University of Minnesota, Austin, MN 55912, USA; 3 Department of Biomedical Sciences, Charles E. Schmidt College of Medicine, Florida Atlantic University, Boca Raton, FL 33431, USA; 4 Department of Parmacognosy, Institute of Biomedical Sciences, Tokushima University Graduate School, Tokushima 770-8503, Japan; 5 Laboratory of Pharmacology, Faculty of Pharmacy, Osaka Ohtani University, Osaka 584-8540, Japan; 6 Medical Corporation Kinshukai, Osaka 558-0011, Japan; 7 Department of Otolaryngology, Institute of Biomedical Sciences, Tokushima University Graduate School, Tokushima 770-8503, Japan

**Keywords:** allergic rhinitis, betuletol, eosinophilia, IL-33, propolis

## Abstract

Propolis, a resinous substance produced by honeybees, has been used in folk medicine since ancient times due to its many biological benefits such as antitumor, antioxidant, antimicrobial, anti-inflammatory, and immunomodulatory effects. Propolis contains flavonoids, terpenoids, aromatic aldehydes, and alcohols, which vary with different climate and environmental conditions. In our study, we examined the antiallergic activity of Brazilian green propolis (BGP) and isolated the active compound that can suppress an allergy-sensitive gene, IL-33, expression and eosinophilia. Ethanolic extract of BGP freeze-dried powder was fractionated with several solvent systems, and the active fractions were collected based on activity measurement. The single active compound was found by thin-layer chromatography. Using column chromatography and NMR, the active compound was isolated and identified as 3,5,7-trihydroxy-6,4’-dimethoxyflavone, also known as betuletol. Further, the antiallergic activity of that has been examined in PMA-induced up-regulation of IL-33 gene expression in Swiss 3T3 cells. Our data showed the IL-33 gene suppression both by BGP and the isolated active compound, betuletol. We also found that betuletol suppressed ERK phosphorylation, suggesting it could be effective in suppressing IL-33 mediated eosinophilic chronic inflammation and will provide new insights to develop potent therapeutics against allergic inflammations.

## 1. Introduction

Allergic rhinitis (AR) is an inflammatory condition of the nasal mucosa characterized by the symptoms of nasal itching, sneezing, anterior nasal secretions, and nasal blockage. Worldwide, more than 600 million persons have AR [1]. Symptoms of AR are divided into two categories: acute and chronic [2]. Eosinophils play a major role in chronic symptoms of allergy [3,4]. Our study indicates that Interleukin (IL)-33 is an allergy-sensitive gene to eosinophilic inflammation and found that the levels of eosinophils were correlatively promoted with nasal mucosal IL-33 mRNA levels in pollinosis patients, indicating that the activation of IL-33 gene expression participates in eosinophilia [5,6]. IL-33 is required in the pathogenesis of AR through the induction of Th2 cytokines and eosinophils and could be controlled by suppressing IL-33 gene expression [7,8]. IL-33 was originally described as a nuclear factor protein in endothelial cells of high endothelial venules [9]. IL-33 is constitutively expressed and localized in the nucleus of epithelial and endothelial cells from various tissues [10,11]. IL-33 can induce Th2 cytokine production in Th2 cells, mast cells, basophils, eosinophils, and recently identified innate immune cells (natural helper cells and neurocytes) [12,13,14,15,16,17,18]. IL-33 can induce Th2 cytokine-mediated allergic inflammation [19]. Antihistamines are the most critical therapeutics for pollinosis and other allergic diseases. However, they are not potent enough to alleviate chronic symptoms, so the development of novel therapeutics is highly needed. Despite still having some side effects, these are applied to the treatment of allergy [20,21], but the mechanism is still unknown. Antiallergic natural medicines having minimum side effects can be a beneficial way for allergic treatment [22,23,24]. Those natural substances are believed to be less toxic than synthetic chemical compounds, and establishing the molecular mechanism may guide us to better develop drugs to treat allergy and allergic complications [25,26]. For that reason, many researchers, including us, have been attempting to isolate and determine the structures of critical compounds in these complicated mixtures and examine their therapeutic utility for allergic diseases including AR. Honey and honey products including propolis, have long been studied for having multiple biological and pharmacological properties, and the mechanisms of action have been extensively investigated in the last years such as antiallergic, anticancer, and anti-inflammatory activity [27,28,29]. Brazilian green propolis (BGP) alleviated the nasal symptoms in allergic rats and was revealed to have a suppressive effect on allergy-sensitive genes such as histamine H_1_ receptor (H1R), IL-4, IL-5, and IL-9 expression in both in vitro and in vivo [30]. Compounds isolated from BGP that suppress IL-33 gene expression may be effective for chronic symptoms.

In this investigation, we isolated and identified the active compound betuletol from BGP that can suppress IL-33 mRNA expression in PMA-induced fibroblasts. It also inhibited the ERK, the signal molecule for IL-33 gene expression, and IL-33-mediated allergic inflammation and eosinophilia. It suggested that betuletol could be a better therapeutic to treat chronic allergic condition. Further, its effect will be investigated in allergic model animals.

## 2. Results

### 2.1. Isolation and Identification of Active Compound from BGP

**Figure 1:**
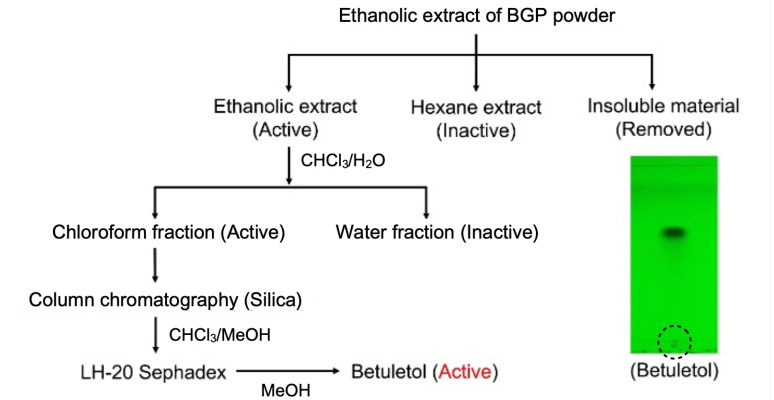
Flowchart of the isolation process for the ethanolic extract of BGP. Active chloroform fraction subjected to silica gel column chromatography eluting with chloroform/methanol gradient solvents. Fractions were further purified by repeated Sephadex LH-20 column chromatography eluting with methanol. The TLC plate was spotted at the position marked with dash circles with purified sample. A single spot was detected on TLC visualized by an UV lamp and identified as betuletol by analysis of ^1^H NMR spectrum. BGP, Brazilian green propolis; TLC, thin layer chromatography.

**Figure 2:**
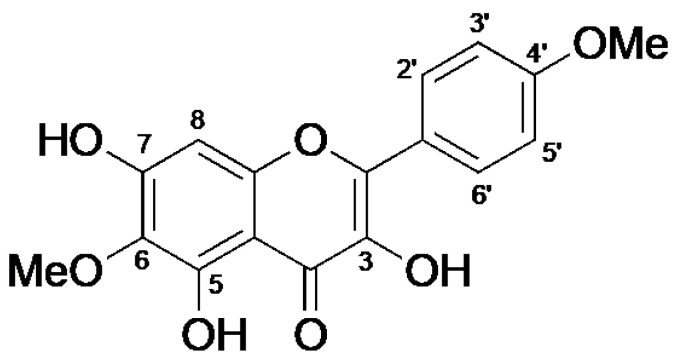
The structure of 3,5,7-trihydroxy-6,4’-dimethoxyflavone (betuletol) isolated from Brazilian green propolis.

As shown in Figure 1, the ethanolic extract of freeze-dried powder of Brazilian green propolis (BGP) was fractionated using various solvents; the suppressive activity of each fraction was examined using PMA-induced up-regulation of IL-33 gene expression in Swiss 3T3 cells using our previously established procedure [5,6]. The chloroform fraction exhibited the highest activity; this fraction was further fractionated by silica gel column chromatography, followed by Sephadex LH-20 column, resulting in the isolation of an active molecule. A single spot found by TLC analysis. Then, we further purified a compound by repeated Sephadex LH-20 column chromatography eluted with MeOH (Figure 1). Several extensive spectroscopic analyses (FTIR, NMR) identified the compound as 5,7-trihydroxy-6,4’-dimethoxyflavone, also known as betuletol (Figure 2, Supplementary Figure S1 and Table S1, [31,32,33]).

### 2.2. Suppression of IL-33 Gene Expression by BGP and Betuletol

**Figure 3:**
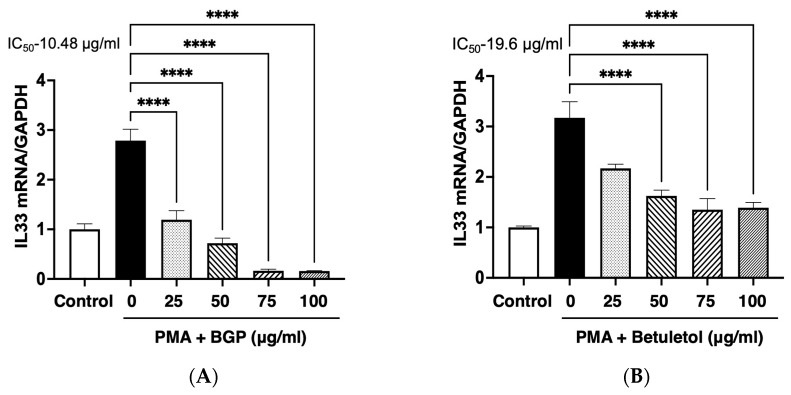
Effects of BGP (**A**) and betuletol (**B**) on PMA-induced up-regulation of IL-33 expression in Swiss 3T3 cells. Swiss 3T3 cells were starved with 0.5% D-MEM medium for 24 h at 37 °C before treatment with 100 nM PMA for 3 h. Swiss 3T3 cells were preincubated with indicated concentrations of BGP (**A**) and betuletol (**B**) for 3 h, respectively. IL-33 mRNA expression was determined using quantitative RT-PCR. Both BGP and betuletol significantly suppressed PMA induced IL-33 gene up-regulation. Data presented as means ± S.E.M.; one-way ANOVA; **** *p* < 0.0001, *n* = 4.

Eosinophil functions can have a significant impact on allergic inflammation. It plays an important role in the pathogenesis of chronic nasal symptoms [34]. IL-33 contributed to the manifestation of chronic allergic symptoms through an increased number of eosinophils [5]. Here, we examined the effect of BGP and betuletol on the IL-33 gene up-regulation that could be closely related to chronic symptoms of AR. We performed real-time RT-PCR with mouse IL-33 primers and a probes kit. In this experiment, the Swiss 3T3 cells were untreated/control, stimulated with PMA (100 nM), and treated with PMA+BGP (25 μg/mL, 50 μg/mL, 75 μg/mL, and 100 μg/mL) (Figure 3A) and PMA + betuletol (10 μg/mL, 20 μg/mL, 40 μg/mL, and 50 μg/mL) (Figure 3B). Results showed that both BGP and betuletol significantly suppressed PMA-induced IL-33 gene up-regulation in a concentration-dependent manner with an IC_50_ value of 10.48 μg/mL and 19.6 μg/mL, respectively (Figure 3).

### 2.3. Inhibition of ERK-1/2 Phosphorylation by Betuletol

**Figure 4:**
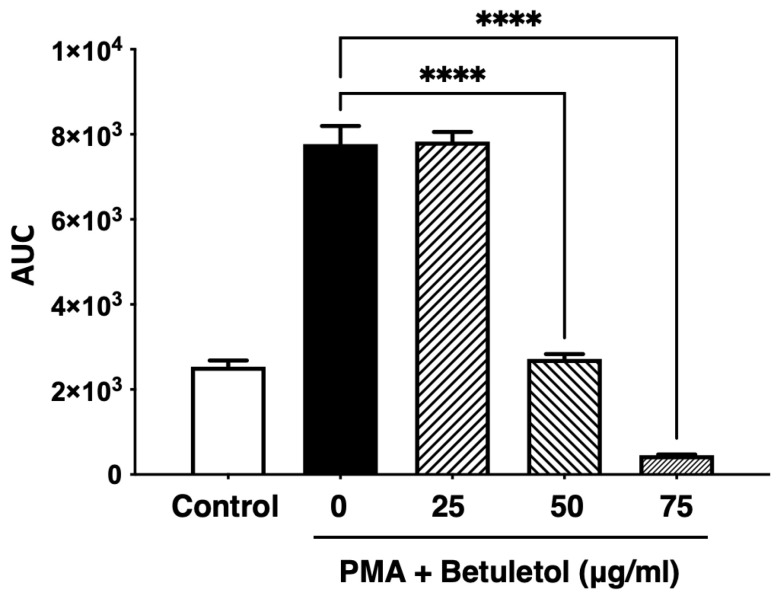
Effect of Betuletol on PMA-induced phosphorylation of ERK. Swiss 3T3 cells were starved with 0.5% FBS medium for 24 h and were then treated with indicated concentrations of betuletol before stimulation with 100 nM PMA for 10 min. Total cell lysates were prepared, and phosphorylation of ERK was determined using immunoblot analysis. Betuletol significantly suppressed PMA-induced ERK phosphorylation. Data presented as means ± S.E.M.; one-way ANOVA; **** *p* < 0.0001, *n* = 4.

We previously reported the involvement of PKCδ/MEK/ERK signaling pathway in inducing inflammatory responses [35]. In this study, to investigate the effect of betuletol in suppressing this inflammatory pathway, we performed Western blot analysis with the PMA-induced Swiss 3T3 cell lysate treated with 25 μg/mL, 50 μg/mL, and 75 μg/mL of betuletol. The immunoblot data showed inhibition of phosphorylation of ERK and calculating the band intensity, and we found that 75 μg/mL of betuletol significantly suppressed the ERK protein expression (Figure 4).

## 3. Discussion

Propolis is generally extracted with ethanol or water, and these extracts have been used in folk medicine. The composition of propolis varies on the solvent used for extraction [29,36]. Additionally, biological activities could be associated with their chemical composition. However, 90–100% ethanolic extract of BGP is frequently used as a healthy food and is reported to have anti-inflammatory effects [37,38]. IL-33 is critical for eosinophil-mediated allergic inflammation, and it was reported that serum levels of IL-33 are significantly elevated in patients with AR [18,39]. Earlier studies described the contribution of IL-33 in eosinophil-, basophil-, and mast cell-mediated allergic inflammation [40].

In this study, the ethanolic extract of BGP was examined and found active against PMA-induced IL-33 mRNA up-regulation in Swiss 3T3 cells (Figure 3A). Further, BGP powder was fractioned with different solvents, and we found maximum suppression of IL-33 mRNA expression in the chloroform fraction. Finally, the active compound was purified from chloroform fraction performing column chromatography, and its structure was identified as betuletol by NMR analysis (Figure 1 and Figure 2, Supplementary Figure S1 and Table S1). Betuletol is found to have anticancer and antihypertensive activity [32,41]. Therefore, we investigated the anti-inflammatory activity of betuletol. Our data showed betuletol suppressed IL-33 gene expression significantly and concentration-dependently (Figure 3B). We have isolated several flavonoids and studied their antiallergic properties, including quercetin, epigallocatechin-3-*O*-gallate from green tea extract [42,43], and (−)-maackiain from Sophora root (*Sophora flavescens*) extract [44]. These compounds suppressed IL-33 gene expression through the inhibition of both PKCδ phosphorylation and followed by ERK phosphorylation with IC_50_ values of ~10 µg/mL [6], but betuletol inhibits only ERK phosphorylation, suggesting a specific and noble mechanism for IL-33 gene suppression. The IC_50_ values of quercetin and maackiain were lower than that of betuletol. However, it is hard to compare these values because target molecules of these compounds were different. Thus, we believe betuletol can improve the symptoms of eosinophilic inflammation more specifically by suppressing IL-33 gene expression and thus decreasing the number of eosinophils.

**Figure 5:**
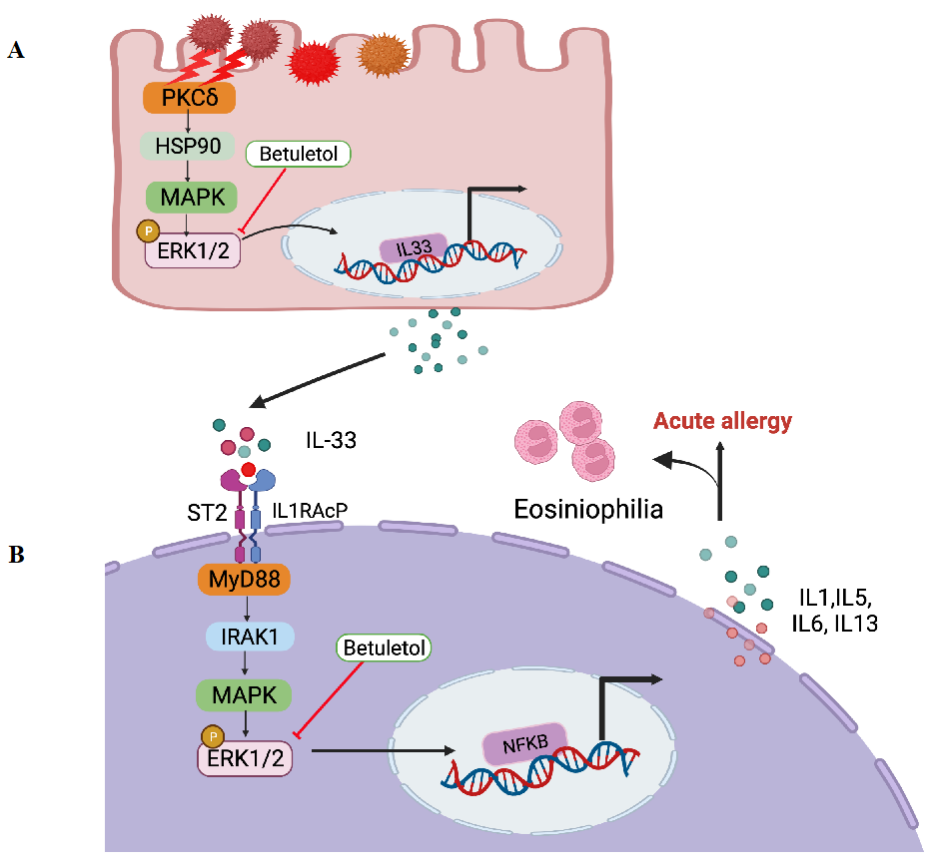
(**A**) Schematic diagram of IL-33 mRNA expression. Pollen/PMA stimulation of epithelial cells causes activation of PKCδ/HSP90/ERK signaling, which causes the expression of the IL-33 gene (**B**) schematic diagram of IL-33/ST2 signaling pathway. IL-33 is the ligand for ST2. It activates the ST2L/IL-1RAcP dimers or is neutralized by binding to sST2. The interaction of IL-33 with ST2 leads to the recruitment of MyD88, which results in the activation of MAPK-ERK and NF-κB, ultimately inducing related gene expression. Inhibition of ERK by betuletol may block the PKCδ/HSP90/ERK-mediated IL-33 mRNA expression and/or, IL-33/ST2-mediated inflammatory reactions. MyD88, myeloid differentiation primary response protein 88; MAPK, mitogen-activated protein kinase; NF-κB, the transcription factor nuclear factor-κB. Illustration created with BioRender.com.

Antihistamine treatment down-regulates the expression of IL-5 mRNA and H1R mRNA but not that of IL-33 mRNA in the mucosa of the respiratory tract in AR [45]. However, flavonoids isolated from Nepalese propolis suppressed IL-33-induced cytokine expression in bone marrow-derived mast cells (BMMC) [46]. In this study, the molecular mechanism of betuletol also has been elucidated. The expression of the IL-33 receptor, interleukin-1 receptor-like 1 (ST2), was identified at the mRNA and protein levels in isolated adult rat cardiac fibroblasts [47]. Investigation of ST2/IL-33 signaling has been implicated in various inflammatory diseases such as cardiac disease, inflammatory bowel diseases, and type 2 diabetes [48,49,50,51]. Upon IL-33 binding, the membrane-anchored ST2 forms a heterodimer along with IL-1RAP leading to the binding of MyD88 and subsequent IL-1R-associated kinase activation, which can initiate mitogen-activated protein kinase (MAPK), extracellular signal-regulated kinase (ERK1/2), and NF-κB pathways [52,53]. We found that betuletol neither suppressed H1R gene expression nor inhibited PKCδ phosphorylation (data not shown) but significantly inhibited the phosphorylation of ERK (Figure 4). These findings suggest that betuletol possibly exerts its antiallergic activities by affecting PKCδ/ERK-mediated IL-33 mRNA expression and/or IL33/ST2-mediated tyrosine phosphorylation of ERK. Therefore, we can summarize the mechanism of action of Betuletol in Figure 5.

Seasonal allergy and AR pathogenesis have recently come into question, given the failure of many pharmaceutical agents in normal humans. Therefore, the pursuit of novel mechanisms mediating AR pathogenesis is of critical importance to future allergic treatment. Betuletol showed a significant suppressive activity against IL-33-mediated inflammation. Moreover, further investigation on the effect of betuletol in allergic model animals will provide more information and direct us to develop new drugs to prevent allergic inflammations.

## 4. Materials and Methods

### 4.1. Materials

Brazilian green propolis ethanolic extract (Lot. No 120928, BGP), standardized to contain 8.0% artepillin C and 0.14% culifolin was provided by Yamada Bee Company, Inc, Japan. Silica gel (N-60) and Sephadex LH-20 were from GE Healthcare (Tokyo, Japan). Precoated TLC plates (0.25 mm, silica gel Kieselgel 60F254 and silica gel 60 RP-18 F254S) were from Nacalai Tesque Inc. (Kyoto, Japan). RNAiso Plus was from Takara Bio Inc. (Kyoto, Japan). Antibodies for ERK (K-23, sc-94), phospho-ERK (E-4, sc-7383) were from Santa Cruz Biotechnology (Santa Cruz, CA, USA). The antibody for β-actin (#4697) was from Cell Signaling Technology Japan (Tokyo, Japan). Goat anti-rabbit IgG (H+L)-HRP conjugate (#170-6515) and Immuno-Star goat anti-mouse HRP conjugate (#170-5047) were from Bio-Rad (Richmond, CA, USA). All other chemicals were of analytical grade.

### 4.2. Propolis Powder

Brazilian green propolis (2 g) was pulverized with a homogenizer and extracted in 20 mL of ethanol (EtOH). After stirring at room temperature for 12 h, the filtrate was concentrated until the solid content reached 55%. The solid content was evaluated after concentration in vacuo and further drying in an oven at 105 °C for 4 h [35].

### 4.3. Fractionation and Isolation

Ethanolic extract of BGP was fractionated with EtOH and hexane mixture. After removing the semisolid insoluble residue, the active ethanolic fraction was further fractionated with a chloroform/water mixture. The chloroform (CHCl_3_) fraction exhibited the highest activity, and this fraction was then fractionated by silica gel (N-60) column chromatography eluted with CHCl_3_: methanol (MeOH) (99:1) followed by 98:2 ratio. A total of 100 fractions (each 5 mL) were collected, and their composition was monitored by thin-layer chromatography (TLC) with CHCl_3_: MeOH (9:1). TLC was performed on precoated plates (0.25 mm, silica gel Kieselgel 60F254 and silica gel 60 RP-18 F254S). All the extract procedures were performed with commercially available organic solvents. A total of 11 fractions were collected and dried. Fraction #1 was further purified by repeated Sephadex LH-20 column chromatography and eluted with MeOH, finally yielding purified compounds.

### 4.4. Structure Identification

Further several spectroscopic methods were performed for structure identification of the single active compound isolated from BGP by Sephadex LH-20 column. ^1^H NMR spectrum was run on a Bruker AVANCE-500 instrument (Hitachi High-Tech, Tokyo, Japan) with CDCl_3_ using tetramethylsilane as an internal standard.

### 4.5. Real-Time Quantitative RT-PCR

Swiss 3T3 cells were cultured at 37 °C under a humidified 5% CO_2_, 95% air atmosphere in minimal essential medium containing 8% fetal calf serum (Sigma, St.Louis, MO, USA) and 1% antibiotic-antimycotic (Nacalai Tesque Inc.). Swiss 3T3 cells cultured to 70% confluency in 6-well dishes were serum-starved for 24 h and treated with reagents 1 h before phorbol-12-myristate-13-acetate (PMA, Sigma) stimulation. After 3h treatment with PMA, the cells were harvested with 700 μL of RNAiso Plus mixed with 140 μL of chloroform and centrifuged at 15,000 rpm for 15 min at 4 °C. The aqueous phase was collected, and RNA was precipitated by the addition of isopropyl alcohol. After centrifugation at 15,000 rpm for 15 min at 4 °C, the resulting RNA pellet was washed with ice-cold 70% ethanol. Total RNA was resolved in 10 μL of diethylpyrocarbonate-treated water, and 5 μL of each RNA sample was used for the reverse transcription reaction. RNA samples were reverse transcribed to cDNA using a High-Capacity cDNA Reverse Transcription kit (Applied Biosystems, Foster City, CA, USA). TaqMan primers and the probe were designed using Primer Express (Applied Biosystems). Real-time PCR was conducted using a GeneAmp 7300 sequence detection system (Applied Biosystems). Mouse IL-33 primers and a probe kit (Mm00505403_m1, Applied Biosystems) were used to determine mouse IL-33 mRNA levels. Rodent GAPDH Control Reagents (Applied Biosystems) were used to standardize the starting material, and the data were expressed as the ratio to GAPDH mRNA.

### 4.6. Immunoblot Analysis

For the immunoblot analysis, 10 μg of each protein sample was separated on a 10% SDS-PAGE gel and then transferred onto a nitrocellulose membrane (Bio-Rad). The membrane was briefly rinsed in Tris-buffered saline containing 0.1% Tween 20 (TBS-T) and then incubated for 1 h at room temperature in TBS-T containing 5% skim milk (Difco, BD Japan, Tokyo, Japan) or 3% BSA (for detecting phosphoproteins; Sigma). The membrane was then incubated with a primary antibody (total ERK (K-23), 1:1000; phospho-ERK (E-4), 1:1000; β-actin (#4697, 1:2000)) overnight at 4 °C. Goat anti-rabbit IgG (H+L)-HRP conjugate (#170-6515, 1:10,000) or Immuno-Star goat anti-mouse HRP conjugate (#170-5047, 1:10,000) was used as the secondary antibody, and proteins were visualized with an Immobilon Western Chemiluminescent HRP substrate (Merk Millipore, Billeria, MA, USA).

### 4.7. Statistical Analysis

The results are shown as the standard error of the mean (S.E.M.). Statistical analyses were performed with unpaired *t*-tests or one-way ANOVA (analysis of variance) with Dunnett’s test using the GraphPad Prism software (GraphPad Software Inc., La Jolla, CA). Values of *p* ˂ 0.05 were considered statistically significant.

## Data Availability

The data presented in this study are available on request from the corresponding author.

## Supplementary Materials

The following supporting information can be downloaded at: https://www.mdpi.com/article/10.3390/molecules27175459/s1, Figure S1: ^1^H NMR spectrum for 3,5,7-trihydroxy-6,4’-dimethoxyflavone (betuletol) measured in CDCl_3_ (500 MHz); Table S1: ^1^H NMR data for 3,5,7-trihydroxy-6,4’-dimethoxyflavone (betuletol) measured in CDCl_3_ (500 MHz).

## References

[1] Bousquet J., Dahl R., Khaltaev N. (2007). Global alliance against chronic respiratory diseases. Allergy.

[2] Skoner D.P. (2001). Allergic rhinitis: Definition, epidemiology, pathophysiology, detection, and diagnosis. J. Allergy Clin. Immunol..

[3] Barnes P.J. (2018). Targeting cytokines to treat asthma and chronic obstructive pulmonary disease. Nat. Rev. Immunol..

[4] Valent P., Degenfeld-Schonburg L., Sadovnik I., Horny H.P., Arock M., Simon H.U., Reiter A., Bochner B.S. (2021). Eosinophils and eosinophil-associated disorders: Immunological, clinical, and molecular complexity. Semin Immunopathol..

[5] Islam R., Mizuguchi H., Shaha A., Nishida K., Yabumoto M., Ikeda H., Fujino H., Kitamura Y., Fukui H., Takeda N. (2018). Effect of wild grape on the signaling of histamine H_1_ receptor gene expression responsible for the pathogenesis of allergic rhinitis. J. Med. Invest..

[6] Nakano S., Yamamoto S., Esu T., Naniwa S., Konishi Y., Wakugawa T., Kitamura Y., Fujii T., Kamimura S., Fukui H. (2021). Effects of Syo-seiryu-to and Its Constituent Crude Drugs on Phorbol Ester-Induced Up-Regulation of IL-33 and Histamine H_1_ Receptor mRNAs in Swiss 3T3 and HeLa Cells. Allergies.

[7] Peebles R.S., Aronica M.A. (2019). Proinflammatory Pathways in the Pathogenesis of Asthma. Clin. Chest Med..

[8] Xu J., Guardado J., Hoffman R., Xu H., Namas R., Vodovotz Y., Xu L., Ramadan M., Brown J., Turnquist H.R. (2017). IL33-mediated ILC2 activation and neutrophil IL5 production in the lung response after severe trauma: A reverse translation study from a human cohort to a mouse trauma model. PLoS Med..

[9] Baekkevold E.S., Roussigne M., Yamanaka T., Johansen F.E., Jahnsen F.L., Amalric F., Brandtzaeg P., Erard M., Haraldsen G., Girard J.P. (2003). Molecular characterization of NF-HEV, a nuclear factor preferentially expressed in human high endothelial venules. Am. J. Pathol..

[10] Carriere V., Roussel L., Ortega N., Lacorre D.A., Americh L., Aguilar L., Bouche G., Girard J.P. (2007). IL-33, the IL-1-like cytokine ligand for ST2 receptor, is a chromatin-associated nuclear factor in vivo. Proc. Natl. Acad. Sci. USA.

[11] Moussion C., Ortega N., Girard J.P. (2008). The IL-1-like cytokine IL-33 is constitutively expressed in the nucleus of endothelial cells and epithelial cells in vivo: A novel ‘alarmin’?. PLoS ONE.

[12] Ho L.H., Ohno T., Oboki K., Kajiwara N., Suto H., Iikura M., Okayama Y., Akira S., Saito H., Galli S.J. (2007). IL-33 induces IL-13 production by mouse mast cells independently of IgE-FcepsilonRI signals. J. Leukoc. Biol..

[13] Kondo Y., Yoshimoto T., Yasuda K., Futatsugi-Yumikura S., Morimoto M., Hayashi N., Hoshino T., Fujimoto J., Nakanishi K. (2008). Administration of IL-33 induces airway hyperresponsiveness and goblet cell hyperplasia in the lungs in the absence of adaptive immune system. Int. Immunol..

[14] Matsuba-Kitamura S., Yoshimoto T., Yasuda K., Futatsugi-Yumikura S., Taki Y., Muto T., Ikeda T., Mimura O., Nakanishi K. (2010). Contribution of IL-33 to induction and augmentation of experimental allergic conjunctivitis. Int. Immunol..

[15] Moro K., Yamada T., Tanabe M., Takeuchi T., Ikawa T., Kawamoto H., Furusawa J., Ohtani M., Fujii H., Koyasu S. (2010). Innate production of T(H)2 cytokines by adipose tissue-associated c-Kit(+)Sca-1(+) lymphoid cells. Nature.

[16] Neill D.R., Wong S.H., Bellosi A., Flynn R.J., Daly M., Langford T.K., Bucks C., Kane C.M., Fallon P.G., Pannell R. (2010). Nuocytes represent a new innate effector leukocyte that mediates type-2 immunity. Nature.

[17] Smithgall M.D., Comeau M.R., Yoon B.R., Kaufman D., Armitage R., Smith D.E. (2008). IL-33 amplifies both Th1- and Th2-type responses through its activity on human basophils, allergen-reactive Th2 cells, iNKT and NK cells. Int. Immunol..

[18] Stolarski B., Kurowska-Stolarska M., Kewin P., Xu D., Liew F.Y. (2010). IL-33 exacerbates eosinophil-mediated airway inflammation. J. Immunol..

[19] Smith D.E. (2010). IL-33: A tissue derived cytokine pathway involved in allergic inflammation and asthma. Clin. Exp. Allergy.

[20] Greiner A.N., Meltzer E.O. (2011). Overview of the treatment of allergic rhinitis and nonallergic rhinopathy. Proc. Am. Thorac. Soc..

[21] Lipworth B.J., Jackson C.M. (2000). Safety of inhaled and intranasal corticosteroids: Lessons for the new millennium. Drug. Saf..

[22] Azab A., Nassar A., Azab A.N. (2016). Anti-Inflammatory Activity of Natural Products. Molecules.

[23] Miguel M.G. (2020). Editorial to Special Issue-Anti-Inflammatory Activity of Natural Products. Molecules.

[24] Yuan G., Wahlqvist M.L., He G., Yang M., Li D. (2006). Natural products and anti-inflammatory activity. Asia Pac. J. Clin. Nutr..

[25] Bernardini S., Tiezzi A., Laghezza Masci V., Ovidi E. (2018). Natural products for human health: An historical overview of the drug discovery approaches. Nat. Prod. Res..

[26] David B., Wolfender J.-L., Dias D.A. (2015). The pharmaceutical industry and natural products: Historical status and new trends. Phytochemistry Rev..

[27] Takeuchi H., Kitano H., Okihara K., Hashimoto K., Enomoto T. (2009). Optimum dose of propolis supplements for the management of Japanese cedar pollinosis: A randomized, double-blind, placebo-controlled trial in 2006. Pharmacometrics.

[28] Takeuchi H., Morizane R., Sakoda T., Hashimoto K., Okihara K., Kitano H., Enomoto T., Yamaguchi H. (2010). Efficacy and safety of supplementary diet containing propolis, bee pollen, and mixed herbal extracts in management of Japanese cedar pollinosis (JCP) ─A randomized, double-blind, placebo-controlled study─Japanese Pharmacology & Therapeutics. Jpn. J. Clin. Pharmacol. Ther..

[29] Viuda-Martos M., Ruiz-Navajas Y., Fernandez-Lopez J., Perez-Alvarez J.A. (2008). Functional properties of honey, propolis, and royal jelly. J. Food Sci..

[30] Shaha A., Mizuguchi H., Kitamura Y., Fujino H., Yabumoto M., Takeda N., Fukui H. (2018). Effect of Royal Jelly and Brazilian Green Propolis on the Signaling for Histamine H1 Receptor and Interleukin-9 Gene Expressions Responsible for the Pathogenesis of the Allergic Rhinitis. Biol. Pharm. Bull..

[31] Hattori H., Okuda K., Murase T., Shigetsura Y., Narise K., Semenza G.L., Nagasawa H. (2011). Isolation, identification, and biological evaluation of HIF-1-modulating compounds from Brazilian green propolis. Bioorg. Med. Chem..

[32] Rubio S., Quintana J., Lopez M., Eiroa J.L., Triana J., Estevez F. (2006). Phenylbenzopyrones structure-activity studies identify betuletol derivatives as potential antitumoral agents. Eur. J. Pharmacol..

[33] Morales G., Sierra P., Mancilla A., Paredes A., Loyola L.A., Gallardo O., Borquez J. (2003). Secondary metabolites from four medical plants from northern Chile: Antimicrobial activity and biotoxicity against *Artemia salina*. J. Chil. Chem. Soc..

[34] Kamakura M. (2011). Royalactin induces queen differentiation in honeybees. Nature.

[35] Mizuguchi H., Terao T., Kitai M., Ikeda M., Yoshimura Y., Das A.K., Kitamura Y., Takeda N., Fukui H. (2011). Involvement of protein kinase Cdelta/extracellular signal-regulated kinase/poly(ADP-ribose) polymerase-1 (PARP-1) signaling pathway in histamine-induced up-regulation of histamine H_1_ receptor gene expression in HeLa cells. J. Biol. Chem..

[36] Tani H., Hasumi K., Tatefuji T., Hashimoto K., Koshino H., Takahashi S. (2010). Inhibitory activity of Brazilian green propolis components and their derivatives on the release of cys-leukotrienes. Bioorg. Med. Chem..

[37] Bhargava P., Mahanta D., Kaul A., Ishida Y., Terao K., Wadhwa R., Kaul S.C. (2021). Experimental Evidence for Therapeutic Potentials of Propolis. Nutrients.

[38] Franchin M., Freires I.A., Lazarini J.G., Nani B.D., da Cunha M.G., Colon D.F., de Alencar S.M., Rosalen P.L. (2018). The use of Brazilian propolis for discovery and development of novel anti-inflammatory drugs. Eur. J. Med. Chem..

[39] Haenuki Y., Matsushita K., Futatsugi-Yumikura S., Ishii K.J., Kawagoe T., Imoto Y., Fujieda S., Yasuda M., Hisa Y., Akira S. (2012). A critical role of IL-33 in experimental allergic rhinitis. J. Allergy Clin. Immunol..

[40] Cherry W.B., Yoon J., Bartemes K.R., Iijima K., Kita H. (2008). A novel IL-1 family cytokine, IL-33, potently activates human eosinophils. J. Allergy Clin. Immunol..

[41] Maruyama H., Sumitou Y., Sakamoto T., Araki Y., Hara H. (2009). Antihypertensive effects of flavonoids isolated from brazilian green propolis in spontaneously hypertensive rats. Biol. Pharm. Bull..

[42] Hattori M., Mizuguchi H., Baba Y., Ono S., Nakano T., Zhang Q., Sasaki Y., Kobayashi M., Kitamura Y., Takeda N. (2013). Quercetin inhibits transcriptional up-regulation of histamine H_1_ receptor via suppressing protein kinase C-delta/extracellular signal-regulated kinase/poly(ADP-ribose) polymerase-1 signaling pathway in HeLa cells. Int. Immunopharmacol..

[43] Matsushita C., Mizuguchi H., Niino H., Sagesaka Y., Masuyama K., Fukui H. (2008). Identification of epigallocatechin-3-O-gallate as an active constituent in tea extract that suppresses transcriptional up-regulations of the histamine H_1_ receptor and interleukin-4 genes. J. Trad Med..

[44] Nariai Y., Mizuguchi H., Ogasawara T., Nagai H., Sasaki Y., Okamoto Y., Yoshimura Y., Kitamura Y., Nemoto H., Takeda N. (2015). Disruption of Heat Shock Protein 90 (Hsp90)-Protein Kinase Cdelta (PKCdelta) Interaction by (-)-Maackiain Suppresses Histamine H_1_ Receptor Gene Transcription in HeLa Cells. J. Biol. Chem..

[45] Kitamura Y., Mizuguchi H., Ogishi H., Kuroda W., Hattori M., Fukui H., Takeda N. (2012). Preseasonal prophylactic treatment with antihistamines suppresses IL-5 but not IL-33 mRNA expression in the nasal mucosa of patients with seasonal allergic rhinitis caused by Japanese cedar pollen. Acta Otolaryngol..

[46] Funakoshi-Tago M., Okamoto K., Izumi R., Tago K., Yanagisawa K., Narukawa Y., Kiuchi F., Kasahara T., Tamura H. (2015). Anti-inflammatory activity of flavonoids in Nepalese propolis is attributed to inhibition of the IL-33 signaling pathway. Int. Immunopharmacol..

[47] Zhu J., Carver W. (2012). Effects of interleukin-33 on cardiac fibroblast gene expression and activity. Cytokine.

[48] Bhardwaj A., Januzzi J.L. (2010). ST2: A novel biomarker for heart failure. Expert Rev. Mol. Diagn..

[49] Ciccone M.M., Cortese F., Gesualdo M., Riccardi R., Di Nunzio D., Moncelli M., Iacoviello M., Scicchitano P. (2013). A novel cardiac bio-marker: ST2: A review. Molecules.

[50] Shimpo M., Morrow D.A., Weinberg E.O., Sabatine M.S., Murphy S.A., Antman E.M., Lee R.T. (2004). Serum levels of the interleukin-1 receptor family member ST2 predict mortality and clinical outcome in acute myocardial infarction. Circulation.

[51] Tung Y.C., Chu P.H. (2014). Soluble ST2: A Novel Prognostic Biomarker of Heart Failure. Acta Cardiol. Sin..

[52] Liew F.Y., Girard J.P., Turnquist H.R. (2016). Interleukin-33 in health and disease. Nat. Rev. Immunol..

[53] Nielsen R., Bustamante C., Clark A.G., Glanowski S., Sackton T.B., Hubisz M.J., Fledel-Alon A., Tanenbaum D.M., Civello D., White T.J. (2005). A scan for positively selected genes in the genomes of humans and chimpanzees. PLoS Biol..

