# Supplementary material for: Betuletol, a Propolis Component, Suppresses IL-33 Gene Expression and Effective against Eosinophilia
Source: Molecules. 2022 Aug 25;27(17):5459. doi: 10.3390/molecules27175459 (PMC9457836; doi:10.3390/molecules27175459)
Supplement: Supplementary file 1 [file molecules-27-05459-s001.zip › molecules-1866185-supplementary.pdf]

molecules

MDPI

*Supplementary Materials*

# Betuletol, a Propolis Component, Suppresses IL-33 Gene Expression and Effective against Eosinophilia

**Aurpita Shaha, Rezwanul Islam, Naonobu Tanaka, Yoshiki Kashiwada, Hiroyuki Fukui, Noriaki Takeda, Yoshiaki Kitamura and Hiroyuki Mizuguchi ***

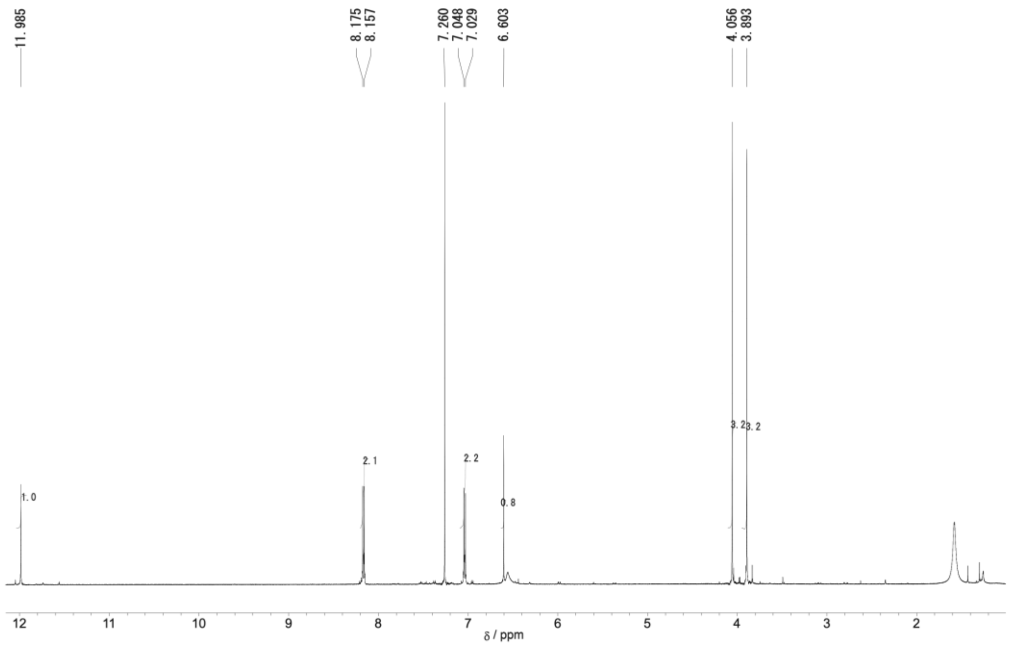


**Figure S1.** $^1$H NMR spectrum for 3,5,7-trihydroxy-6,4'-dimethoxyflavone (betuletol) measured in $CDCl_3$ (500 MHz).

**Table S1.** $^1$H NMR data for 3,5,7-trihydroxy-6,4'-dimethoxyflavone (betuletol) measured in $CDCl_3$ (500 MHz).

| position | $\delta_H$ (*J* in Hz) |
|---|---|
| 8 | 6.60 (1H, s) |
| 2' | 8.16 (1H, d, 8.0) |
| 3' | 7.04 (1H, d, 8.0) |
| 5' | 7.04 (1H, d, 8.0) |
| 6' | 8.16 (1H, d, 8.0) |
| 5-OH | 11.99 (1H, s) |
| 6-OMe | 3.89 (3H, s)[a] |
| 4'-OMe | 4.06 (3H, s)[a] |

[a]Signals maybe interchangeable
